# Supplementary material for: Distinct mechanisms of non-autonomous UPRER mediated by GABAergic, glutamatergic, and octopaminergic neurons
Source: bioRxiv. 2024 Dec 24:2024.05.27.595950. Originally published 2024 May 30. Preprint. [Version 2] doi: 10.1101/2024.05.27.595950 (PMC11160609; doi:10.1101/2024.05.27.595950)
Supplement: Supplement 3 [file media-3.docx]

| **Primer Name** | **Primer Sequence** | **Primer Purpose** |
| --- | --- | --- |
| xbp-1s RT-qPCR Forward | CGTGCCTTTGAATCAGCAGTG | Measurement of xbp-1s transcripts |
| xbp-1s RT-qPCR Reverse | CGAGGTGTCCATCTTCTTGTT | Measurement of xbp-1s transcripts |
| Y45F10D.4 RT-qPCR Forward | AAGCGTCGGAACAGGAATC | Housekeeping gene |
| Y45F10D.4 RT-qPCR Reverse | TTTTTCCGTTATCGTCGACTC | Housekeeping gene |
| sap-49 RT-qPCR Forward | TGGCGGATCGTCGTGCTTCC | Housekeeping gene |
| sap-49 RT-qPCR Reverse | ACGAGTCTCCTCGTTCGTCCCA | Housekeeping gene |
