# Supplementary material for: Distinct mechanisms of non-autonomous UPRER mediated by GABAergic, glutamatergic, and octopaminergic neurons
Source: bioRxiv. 2024 Dec 24:2024.05.27.595950. Originally published 2024 May 30. Preprint. [Version 2] doi: 10.1101/2024.05.27.595950 (PMC11160609; doi:10.1101/2024.05.27.595950)
Supplement: Supplement 8 [file NIHPP2024.05.27.595950v2-supplement-8.pdf]

bioRxiv preprint doi: https://doi.org/10.1101/2024.05.27.595950; this version posted December 24, 2024. The copyright holder for this preprint (which was not certified by peer review) is the author/funder, who has granted bioRxiv a license to display the preprint in perpetuity. It is made available under aCC-BY 4.0 International license.

Supporting Information

Fig. S1. A comparison of pan-neuronal *xbp-1s* expression via two different promoters reveals differences in whole-body transcriptomic changes. Volcano plots of whole-body genome-wide changes in gene expression upon pan-neuronal *xbp-1s* overexpression driven by **(A)** *rab-3* promoter or **(B)** *rgef-1* promoter. Red dots indicate significantly differentially expressed genes with p-value ≤ 0.01. See **Table S3** for a list of differentially expressed genes and expression values. **(C)** Comparison of differentially expressed genes (p-value ≤ 0.01) between worms expressing *xbp-1s* pan-neuronally driven by *rab-3p* or *rgef-1p*. For a complete list of differentially expressed genes in each group, see **Table S4**. **(D)** Heat map of common differentially expressed genes upon pan-neuronal *xbp-1s* expression under control of *rab-3* or *rgef-1* promoter. Warmer colors indicate increased expression, and cooler colors indicate decreased expression. See **Table S5** for a list of genes and values. **(E)** Heat map of UPR^{ER} related gene (GO:0030968) expression under pan-neuronal *xbp-1s* driven by *rab-3* or *rgef-1* promoter. Warmer colors indicate increased expression, and cooler colors indicate decreased expression. See **Table S5** for a list of genes and values. **(F)** Heat map of XBP-1s target gene [31] expression under pan-neuronal *xbp-1s* driven by *rab-3* or *rgef-1* promoter. Warmer colors indicate increased expression, and cooler colors indicate decreased expression. See **Table S5** for a list of genes and values.

Figure S2. *xbp-1s* overexpression in *C. elegans* glutamatergic, octopaminergic, and GABAergic neurons drives differential gene expression. Comparison of differentially expressed genes (p-value ≤ 0.01) in neuronal *xbp-1s* driven by *rgef-1p* and **(A)** glutamatergic (*eat-4p*), **(B)** octopaminergic *(tbh-1p*), and **(C)** GABAergic (*unc-25p*) *xbp-1s* expression. For a complete list of differentially expressed genes in each group, see **Table S3**. Comparison of differentially expressed genes (p-value ≤ 0.01) in glutamatergic, octopaminergic, GABAergic,

bioRxiv preprint doi: https://doi.org/10.1101/2024.05.27.595950; this version posted December 24, 2024. The copyright holder for this preprint (which was not certified by peer review) is the author/funder, who has granted bioRxiv a license to display the preprint in perpetuity. It is made available under aCC-BY 4.0 International license.

and **(D)** dopaminergic or **(E)** serotonergic neurons. See **Table S4**. Gene expression changes in groups of genes related to **(F)** UPR^{ER} (GO:0030968), **(G)** XBP-1s targets [31], **(H)** mitochondrial unfolded protein response (GO:0034514), **(I)** heat shock response (GO:0009408), **(J)** oxidative stress response (GO:0006979), and **(K)** translation (GO:0006412). Significance was determined using a one-sample Wilcoxon test. * = $p \leq 0.05$, ** = $p \leq 0.01$, *** = $p \leq 0.001$, **** = $p \leq 0.0001$, ns = $p > 0.05$

Fig. S3. Glutamatergic, octopaminergic, and GABAergic *xbp-1s* does not increase healthspan. Measurements of fecundity of control (blue) and **(A)** glutamatergic *xbp-1s* (green, *eat-4p*), **(B)** octopaminergic *xbp-1s* (yellow, *tbh-1p*), and **(C)** GABAergic *xbp-1s* (pink, *unc-25p*) animals. Total number of eggs that hatched were counted per animal. Measurements of thrashing of control (blue) and **(D)** glutamatergic *xbp-1s* (green, *eat-4p*), **(E)** octopaminergic *xbp-1s* (yellow, *tbh-1p*), and **(F)** GABAergic *xbp-1s* (pink, *unc-25p*) animals. Number of thrashes was assessed over a 10 second period in animals at day 1 adult (young), day 4-5 adult (middle), and day 9 adult (old) in M9 solution with each thrash being counted as a movement from a concave to a convex formation. For SuperPlots, each small dot represents a single animal with various intensities of colors representing independent biological replicates and each large dot is the median value of each biological replicate. Lines represent the median across all biological replicates and whiskers indicate interquartile range. Statistical analysis was performed using a Mann-Whitney test.

Fig. S4. Glutamatergic, octopaminergic, and GABAergic *xbp-1s* does not alter ER secretory capacity. (A) Representative fluorescent micrographs of day 3 adult animals of VIT2::GFP in control, glutamatergic *xbp-1s* (*eat-4p*), octopaminergic *xbp-1s* (*tbh-1p*), or GABAergic *xpb-1s* (*unc-25p*). All images are contrast-matched. **(B)** Representative fluorescent micrographs of eggs collected using a standard bleaching protocol of day 3 adult animals of VIT2::GFP in control, glutamatergic *xbp-1s* (*eat-4p*), octopaminergic *xbp-1s* (*tbh-1p*), or GABAergic *xpb-1s* (*unc-25p*). All images are contrast-matched. Scale bar represents 500 µm. **(C)** Quantification of eggs from (B) using measurements of integrated intensity. For SuperPlots, each small dot represents a single animal with various intensities of colors representing independent biological replicates and each large dot is the median value of each biological replicate. Lines represent the median across all biological replicates and whiskers indicate interquartile range. Statistical analysis was performed using a Mann-Whitney test.

Fig. S5. Octopaminergic *xbp-1s* promotes lifespan in polyQ40 expression animals, but not on tunicamycin. (A) Lifespan measurements of control (here, *vha-6p::polyQ*), blue), glutamatergic *xbp-1s* animals (*eat-4p*, green), octopaminergic *xbp-1s* separated for normal sized (*tbh-1,* yellow) or stunted growth (*tbh-1,* small, purple), and GABAergic *xbp-1s* (*unc-25p,* pink) animals expressing polyQ40::YFP in the intestine (*vha-6p::polyQ*). **(B)** Median lifespan measurements from 5 replicates of wild-type N2 animals either expressing *vha-6p::polyQ40* (light blue, *vha-6p::polyQ*) or not (dark blue, control). Dots indicate each biological replicate and lines represent median plus interquartile range. **(C)** Lifespan measurements of control (blue, light blue), glutamatergic *xbp-1s* animals (*eat-4p*, green), and GABAergic *xbp-1s* (*unc-25p,* pink) animals moved onto 25 mg/mL tunicamycin (TM) plates starting from day 1 of adulthood. Lifespans were scored every 2 days and data is representative of 3 biological replicates (N). **(D)** Lifespan measurements of control (blue) and octopaminergic *xbp-1s* animals moved onto 25 mg/mL tunicamycin (TM) plates starting from day 1 of adulthood. Octopaminergic *xbp-1s* animals were separated into normal size (yellow, *tbh-1p*) and stunted growth (purple, *tbh-1p,* small). Sample size (n) is written next to each condition followed by significance measured

bioRxiv preprint doi: https://doi.org/10.1101/2024.05.27.595950; this version posted December 24, 2024. The copyright holder for this preprint (which was not certified by peer review) is the author/funder, who has granted bioRxiv a license to display the preprint in perpetuity. It is made available under aCC-BY 4.0 International license.

using Log-Rank testing: n.s. = not significant, * = $p < 0.05$, *** = $p < 0.001$. All statistical analysis is available in **Table S7**.

Table S1. Strains used in this study.

Table S2. Primers used in this study.

Table S3. List of differentially expressed genes.

Table S4. List of all genes in Venn Diagrams.

Table S5. List of all genes used in heat maps.

Table S6. List of all gene ontologies.

Table S7. Lifespan statistics.

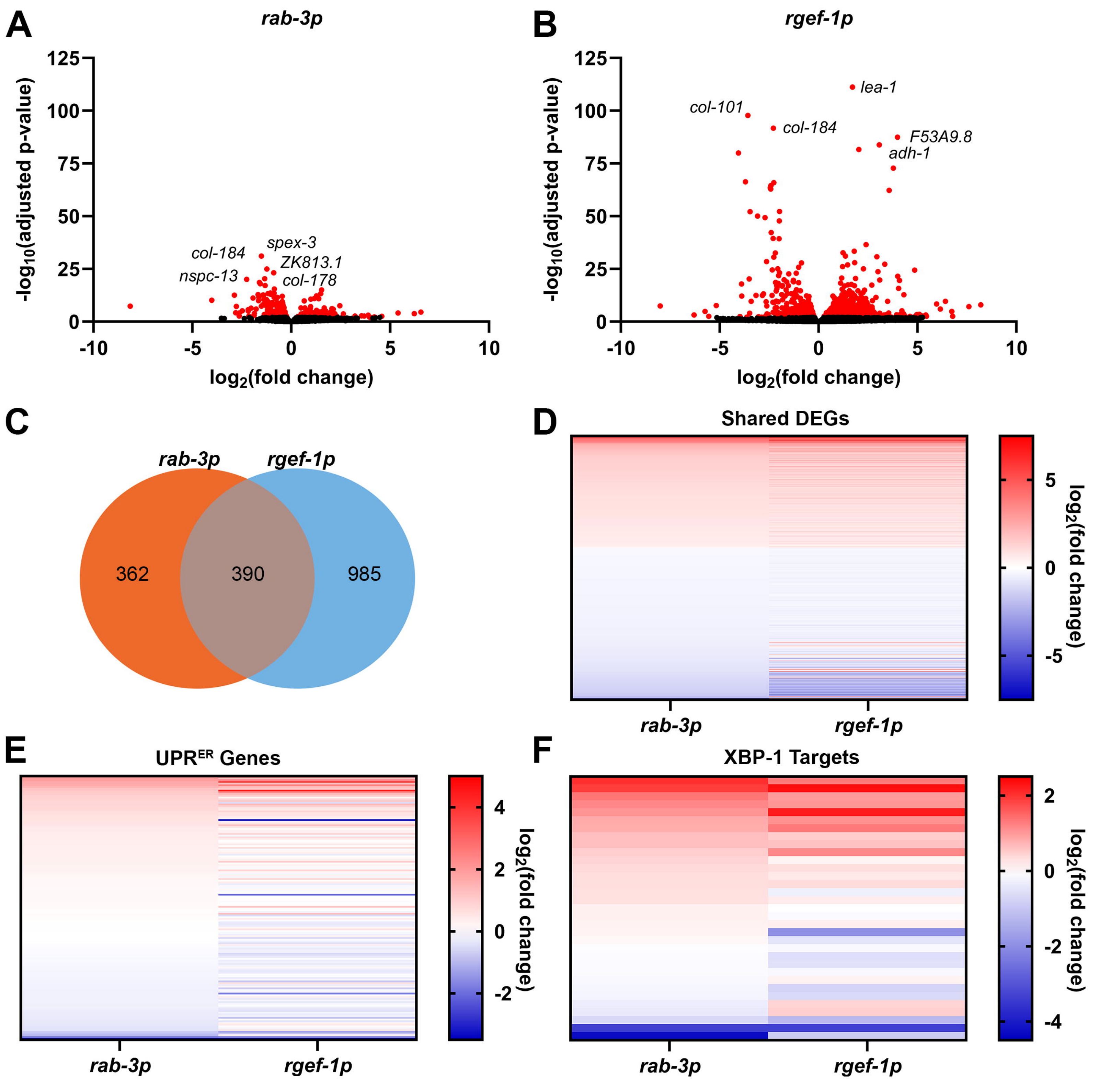

A
rab-3p
-log10(adjusted p-value)
log2(fold change)
col-184
nspc-13
spex-3
ZK813.1
col-178
B
rgef-1p
lea-1
col-101
col-184
F53A9.8
adh-1
C
rab-3p
rgef-1p
362
390
985
D
Shared DEGs
E
UPRER Genes
F
XBP-1 Targets

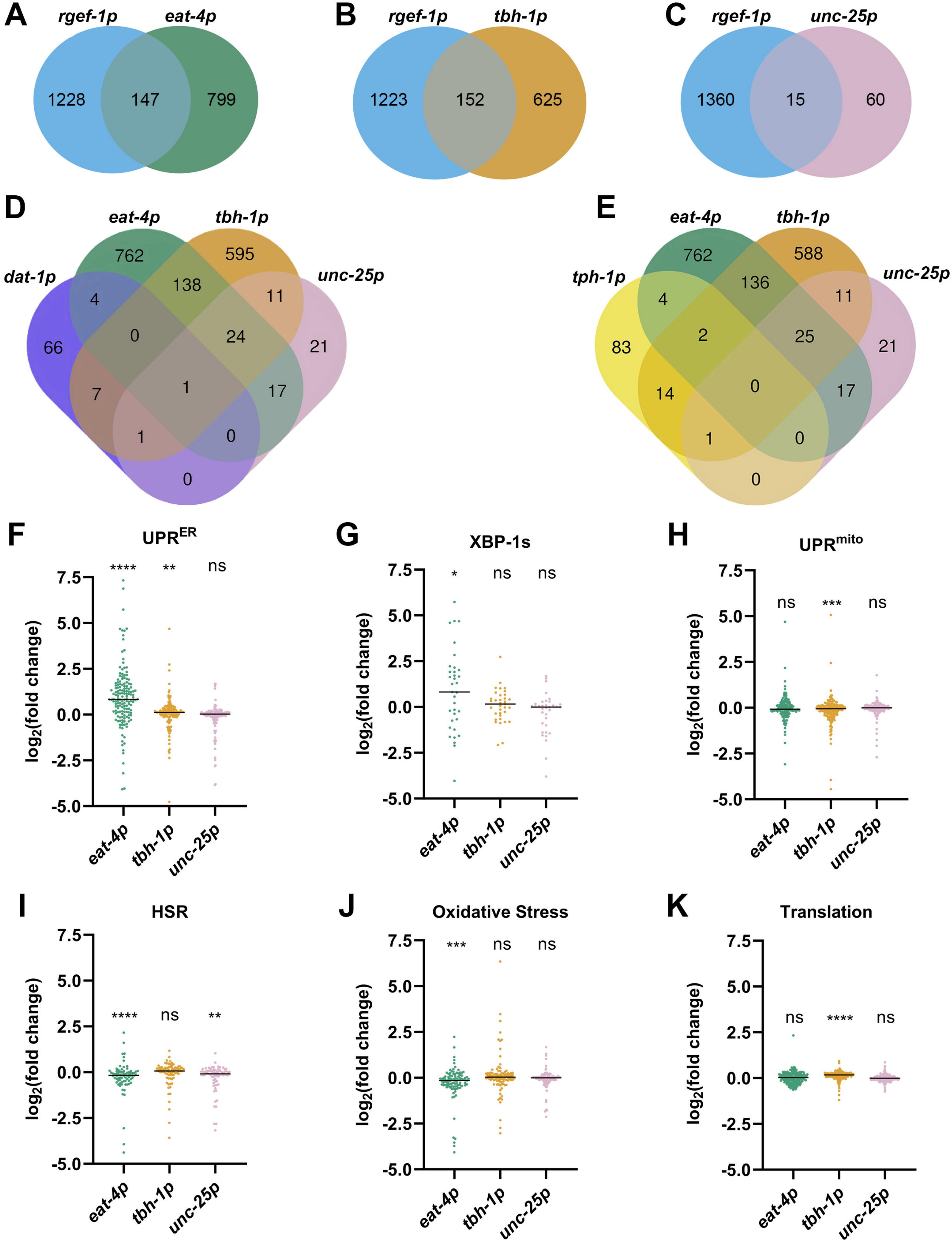
A
rgef-1p
eat-4p
1228
147
799
B
rgef-1p
tbh-1p
1223
152
625
C
rgef-1p
unc-25p
1360
15
60
D
eat-4p
tbh-1p
dat-1p
unc-25p
762
595
138
4
11
66
0
24
21
7
1
17
1
0
0
E
eat-4p
tbh-1p
tph-1p
unc-25p
762
588
136
4
11
83
2
25
21
14
0
17
1
0
0
F
UPR^ER
G
XBP-1s
H
UPR^mito
I
HSR
J
Oxidative Stress
K
Translation
log2(fold change)
eat-4p
tbh-1p
unc-25p
ns
*
**
***
****

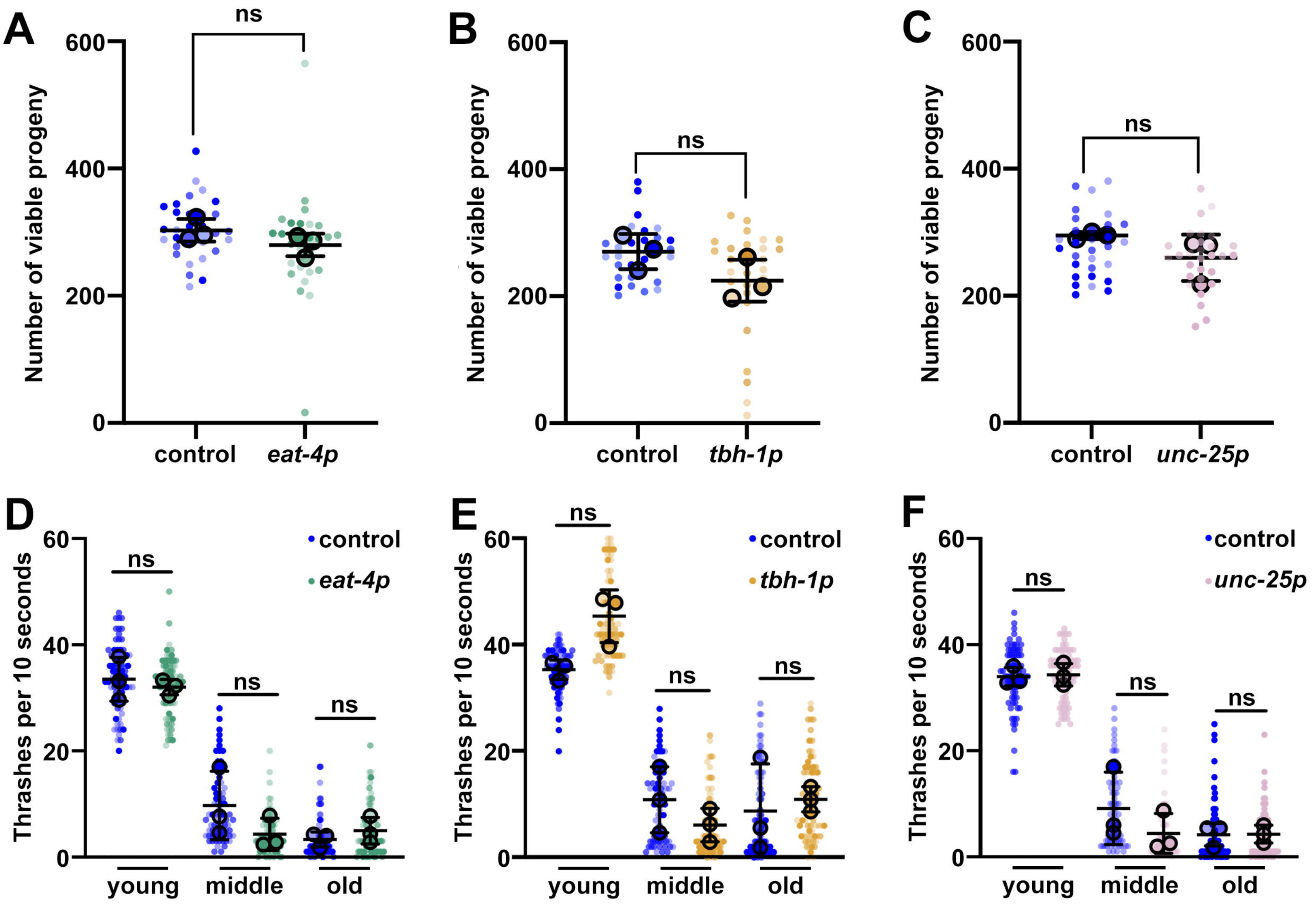

A
B
C
D
E
F
Number of viable progeny
600
400
200
0
ns
control
eat-4p
tbh-1p
unc-25p
Thrashes per 10 seconds
60
40
20
0
young
middle
old

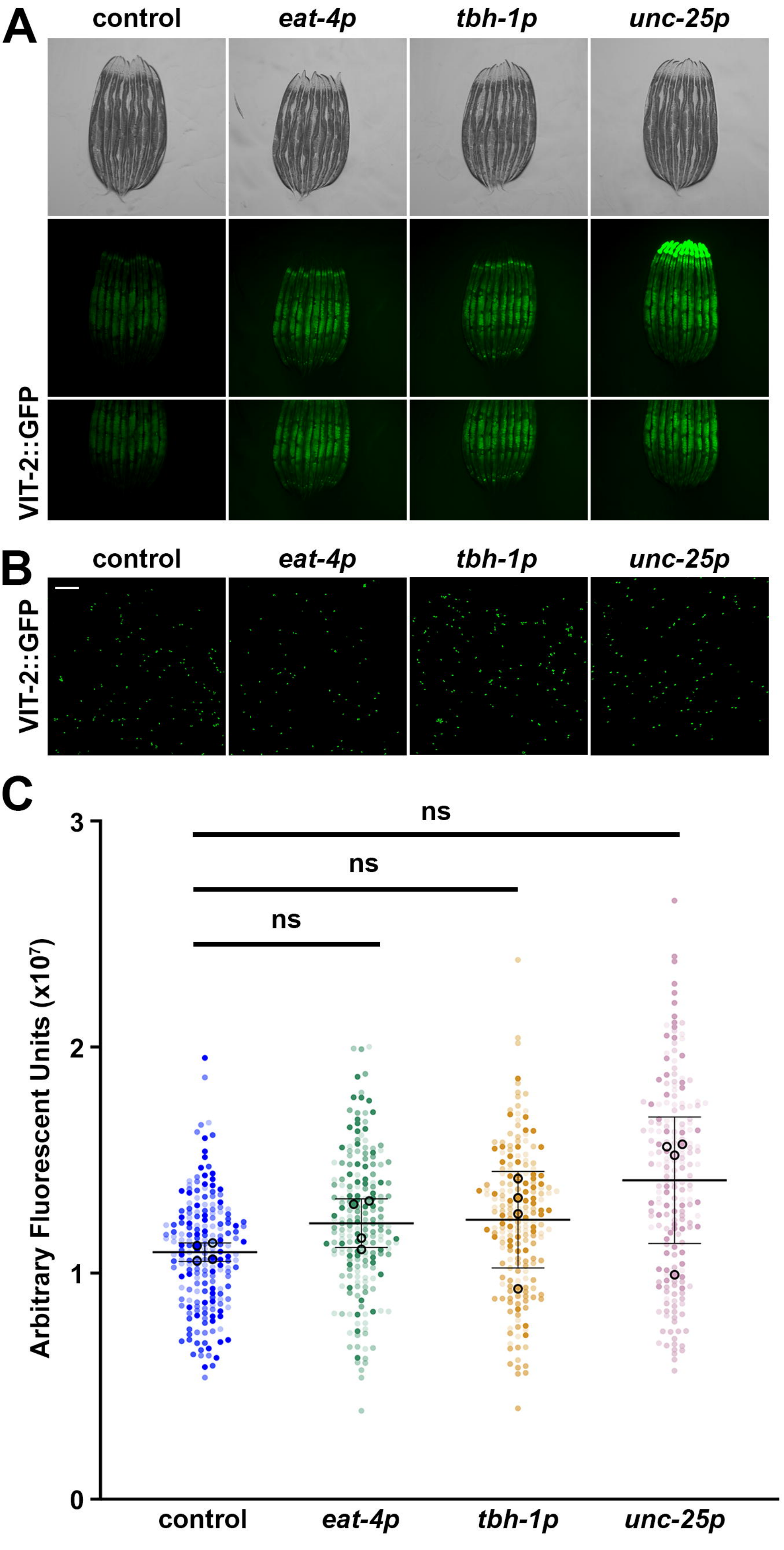

A
control
eat-4p
tbh-1p
unc-25p
VIT-2::GFP
B
control
eat-4p
tbh-1p
unc-25p
VIT-2::GFP
C
ns
ns
ns
Arbitrary Fluorescent Units (x10⁷)
3
2
1
0
control
eat-4p
tbh-1p
unc-25p

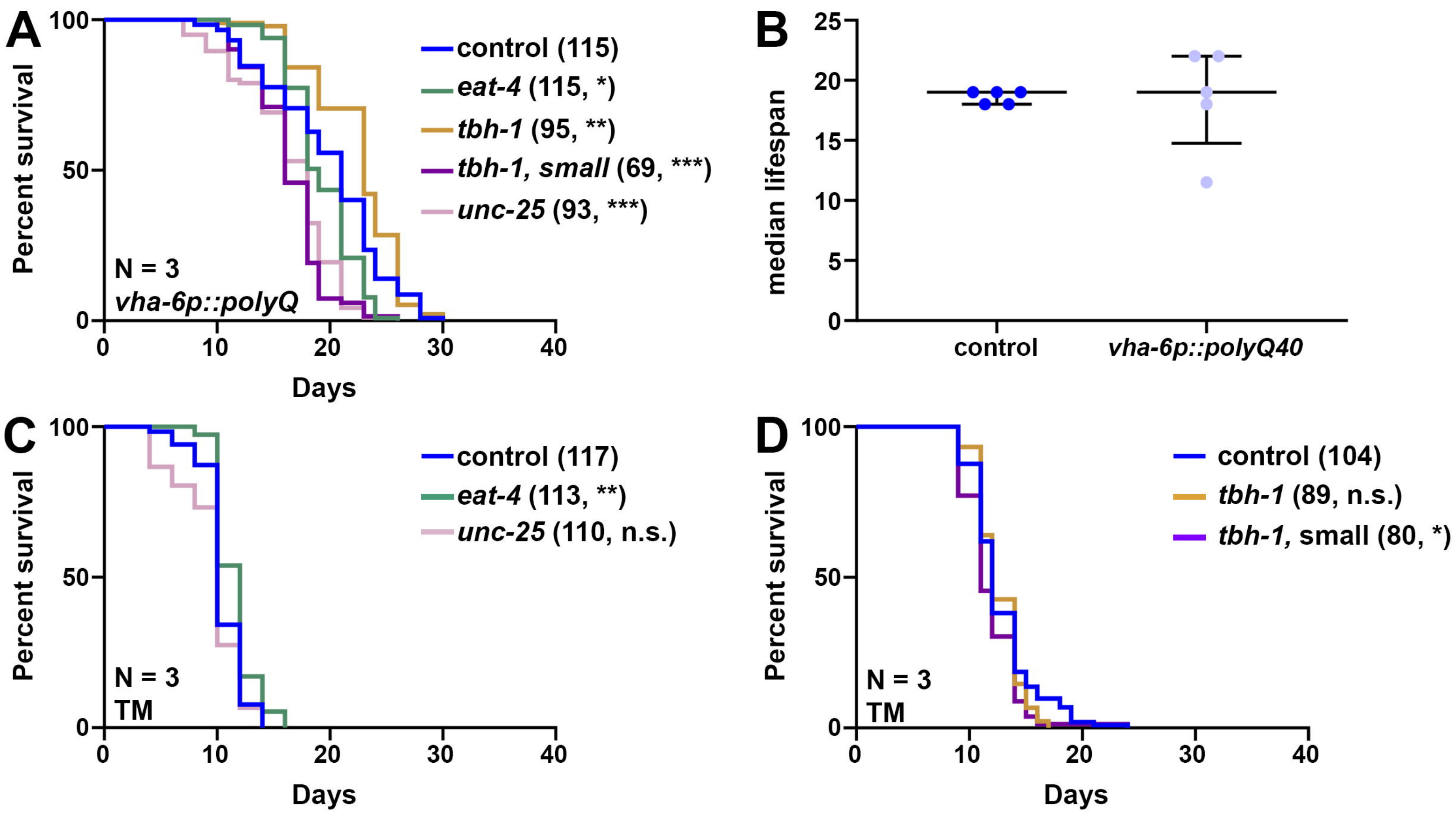
A
B
C
D
Percent survival
Days
control (115)
eat-4 (115, *)
tbh-1 (95, **)
tbh-1, small (69, ***)
unc-25 (93, ***)
N = 3
vha-6p::polyQ
median lifespan
control
vha-6p::polyQ40
control (117)
eat-4 (113, **)
unc-25 (110, n.s.)
N = 3
TM
control (104)
tbh-1 (89, n.s.)
tbh-1, small (80, *)
N = 3
TM
