# Supplementary material for: Distinct mechanisms of non-autonomous UPRER mediated by GABAergic, glutamatergic, and octopaminergic neurons
Source: bioRxiv. 2024 May 30:2024.05.27.595950. Preprint. [Version 1] doi: 10.1101/2024.05.27.595950 (PMC11160609; doi:10.1101/2024.05.27.595950)
Supplement: Supplementary file 1 [file NIHPP2024.05.27.595950v1-supplement-6.pdf]

bioRxiv preprint doi: https://doi.org/10.1101/2024.05.27.595950; this version posted May 30, 2024. The copyright holder for this preprint (which was not certified by peer review) is the author/funder, who has granted bioRxiv a license to display the preprint in perpetuity. It is made available under aCC-BY 4.0 International license.

Supplemental Figure Legends

Fig. S1. *xbp-1s* overexpression in *C. elegans* glutamatergic, octopaminergic, and dopaminergic neurons. (A) Schematic of each neuronal type explored in this study: glutamatergic, *eat-4p* (n = 79, location = head, pharynx, ventral nerve cord and body, tail), octopaminergic, *tbh-1p* (n = 2, location = head), GABAergic, *unc-25p* (n = 32, location = head, ventral nerve cord and body, tail). **(B)** qPCR of transcripts of control (blue), glutamatergic *xbp-1s* (green), octopaminergic *xbp-1s* (yellow), and GABAergic *xbp-1s* (pink) animals grown on empty vector from hatch. RNA was isolated in day 1 adults and data is compared using a standard curve with data represented as relative fold change against control. Each dot represents a biological replicate averaged across three technical replicates per sample. Lines represent geometric mean with geometric standard deviations.

Fig S2. (A) Comparison of differentially expressed genes (p-value ≤ 0.01) in neuronal *xbp-1s* driven by *rgef-1p* or *rab-3p*. Comparison of differentially expressed genes (p-value ≤ 0.01) in glutamatergic, octopaminergic, GABAergic *xbp-1s* and **(B)** neuronal *xbp-1s* driven by *rgef-1p*, **(C)** serotonergic *xbp-1s* driven by *tph-1p*, and **(D)** dopaminergic *xbp-1s* driven by *dat-1p*. See **Table S1**. Heat map of **(E)** lipid homeostasis (GO:0055088) or **(F)** immune response (GO:0006955) gene expression under pan-neuronal, glutamatergic, octopaminergic, and GABAergic *xbp-1s* overexpression. Warmer colors indicate increased expression, and cooler colors indicate decreased expression. See **Table S3**.

Fig. S3. Glutamatergic, octopaminergic, and GABAergic *xbp-1s* does not extend lifespan. Lifespan measurements of control (blue) and **(A)** glutamatergic *xbp-1s* (green, *eat-4p*), **(B)** octopaminergic *xbp-1s* (yellow, *tbh-1p*), **(C)** and GABAergic *xbp-1s* (pink, *unc-25p*) animals. Lifespans were scored every 2 days and data is representative of 3 biological replicates. All statistical analysis is available in **Table S5**.

Fig. S4. Glutamatergic, octopaminergic, and GABAergic *xbp-1s* does not increase healthspan. Measurements of fecundity of control (blue) and **(A)** glutamatergic *xbp-1s* (green, *eat-4p*), **(B)** octopaminergic *xbp-1s* (yellow, *tbh-1p*), and **(C)** GABAergic *xbp-1s* (pink, *unc-25p*) animals. Total number of eggs that hatched were counted per animal. Measurements of thrashing of control (blue) and **(D)** glutamatergic *xbp-1s* (green, *eat-4p*), **(E)** octopaminergic *xbp-1s* (yellow, *tbh-1p*), and **(F)** GABAergic *xbp-1s* (pink, *unc-25p*) animals. Number of thrashes was assessed over a 10 second period in animals at day 1 adult (young), day 4-5 adult (middle), and day 9 adult (old) in M9 solution with each thrash being counted as a movement from a concave to a convex formation. For SuperPlots, each small dot represents a single animal with various intensities of colors representing independent biological replicates and each large dot is the median value of each biological replicate. Lines represent the median across all biological replicates and whiskers indicate interquartile range. Statistical analysis was performed using a Mann-Whitney test.

Fig. S5. Glutamatergic, octopaminergic, and GABAergic *xbp-1s* does not alter ER morphology. (A) Representative fluorescent micrographs of day 3 adult mRuby::HDEL of control, glutamatergic *xbp-1s* (*eat-4p*), and GABAergic *xbp-1s* (*unc-25p*), animals. Images are representative of three independent biological replicates and are independently contrast enhanced for each individual image. Scale bar represents 10 μm. **(B)** Representative fluorescent micrographs of day 3 adult mCherry::HDEL of control and octopaminergic *xbp-1s* (*tbh-1p*), animals. Images are representative of three independent biological replicates and are independently contrast enhanced for each individual image. Scale bar represents 10 μm.

bioRxiv preprint doi: https://doi.org/10.1101/2024.05.27.595950; this version posted May 30, 2024. The copyright holder for this preprint (which was not certified by peer review) is the author/funder, who has granted bioRxiv a license to display the preprint in perpetuity. It is made available under aCC-BY 4.0 International license.

Fig. S6. Glutamatergic, octopaminergic, and GABAergic *xbp-1s* does not alter ER secretory capacity. (A) Representative fluorescent micrographs of day 3 adult animals of VIT2::GFP in control, glutamatergic *xbp-1s* (*eat-4p*), octopaminergic *xbp-1s* (*tbh-1p*), or GABAergic *xpb-1s* (*unc-25p*). All images are contrast matched. **(B)** Representative fluorescent micrographs of eggs collected using a standard bleaching protocol of day 3 adult animals of VIT2::GFP in control, glutamatergic *xbp-1s* (*eat-4p*), octopaminergic *xbp-1s* (*tbh-1p*), or GABAergic *xpb-1s* (*unc-25p*). All images are contrast matched. Scale bar represents 500 µm. **(C)** Quantification of eggs from (B) using measurements of integrated intensity. For SuperPlots, each small dot represents a single animal with various intensities of colors representing independent biological replicates and each large dot is the median value of each biological replicate. Lines represent the median across all biological replicates and whiskers indicate interquartile range. Statistical analysis was performed using a Mann-Whitney test.

Supplementary Tables:

Table S1. List of DEGs.
Table S2. Venn Diagram Gene Names.
Table S3. Heat Maps.
Table S4. GO Biological Process.
Table S5. Lifespan Statistics.

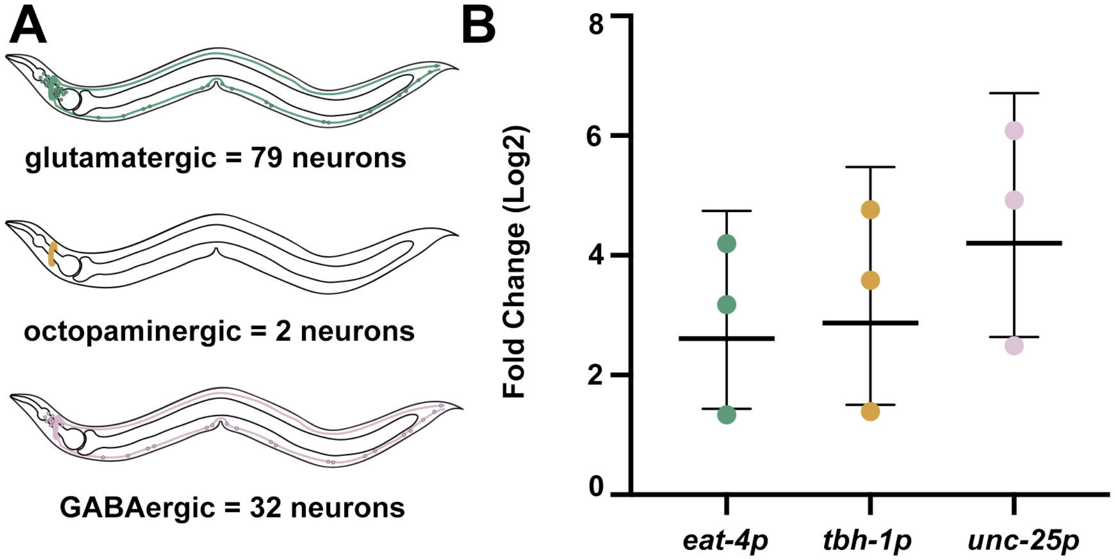
A
glutamatergic = 79 neurons
octopaminergic = 2 neurons
GABAergic = 32 neurons
B
Fold Change (Log2)
0
2
4
6
8
eat-4p
tbh-1p
unc-25p

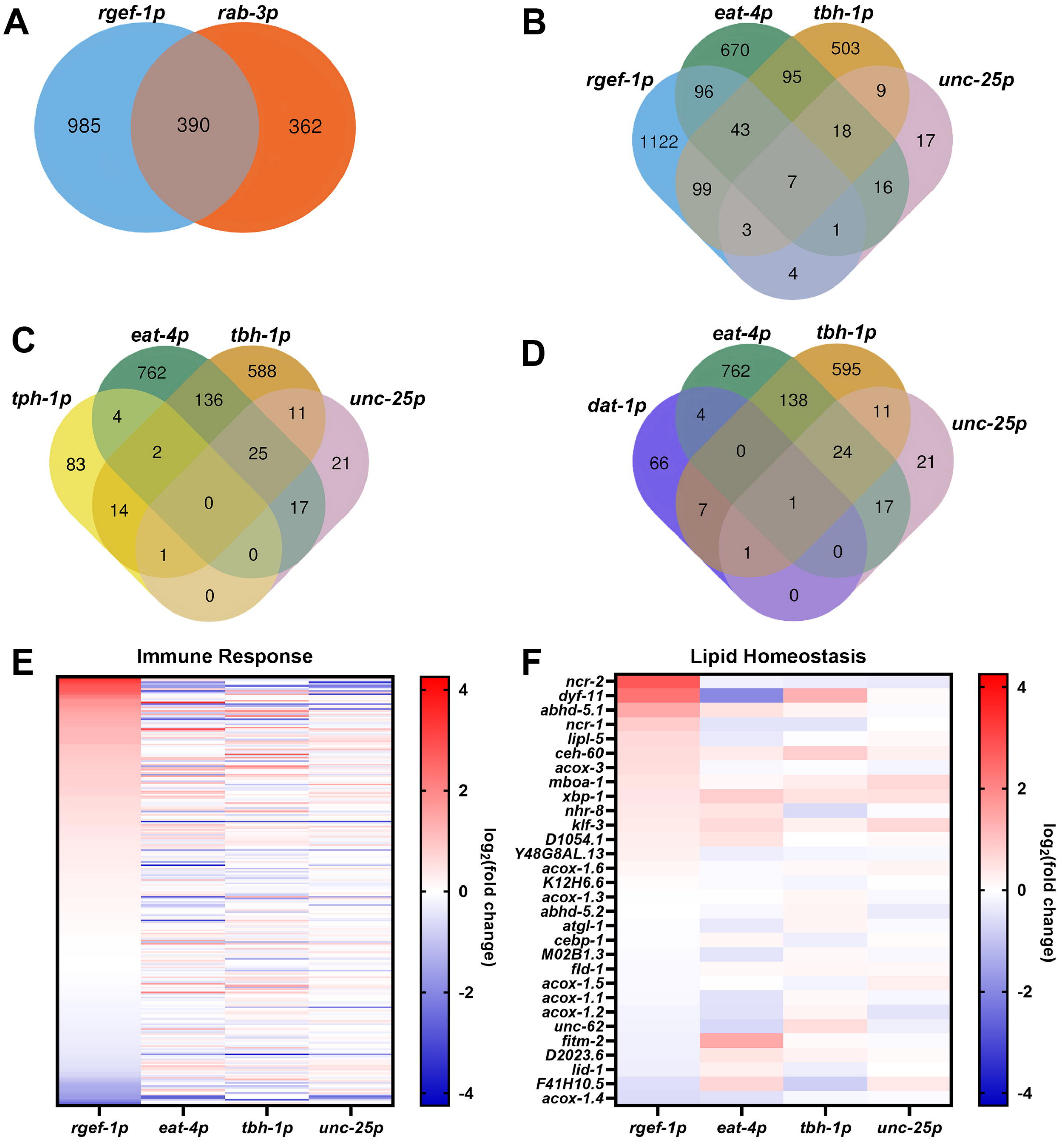
A
rgef-1p
rab-3p
985
390
362
B
eat-4p
tbh-1p
rgef-1p
unc-25p
670
503
96
95
9
1122
43
18
17
99
7
16
3
1
4
C
eat-4p
tbh-1p
tph-1p
unc-25p
762
588
4
136
11
83
2
25
21
14
0
17
1
0
0
D
eat-4p
tbh-1p
dat-1p
unc-25p
762
595
4
138
11
66
0
24
21
7
1
17
1
0
0
E
Immune Response
rgef-1p
eat-4p
tbh-1p
unc-25p
log2(fold change)
4
2
0
-2
-4
F
Lipid Homeostasis
ncr-2
dyf-11
abhd-5.1
ncr-1
lipl-5
ceh-60
acox-3
mboa-1
xbp-1
nhr-8
klf-3
D1054.1
Y48G8AL.13
acox-1.6
K12H6.6
acox-1.3
abhd-5.2
atgl-1
cebp-1
M02B1.3
fld-1
acox-1.5
acox-1.1
acox-1.2
unc-62
fitm-2
D2023.6
lid-1
F41H10.5
acox-1.4
rgef-1p
eat-4p
tbh-1p
unc-25p
log2(fold change)
4
2
0
-2
-4

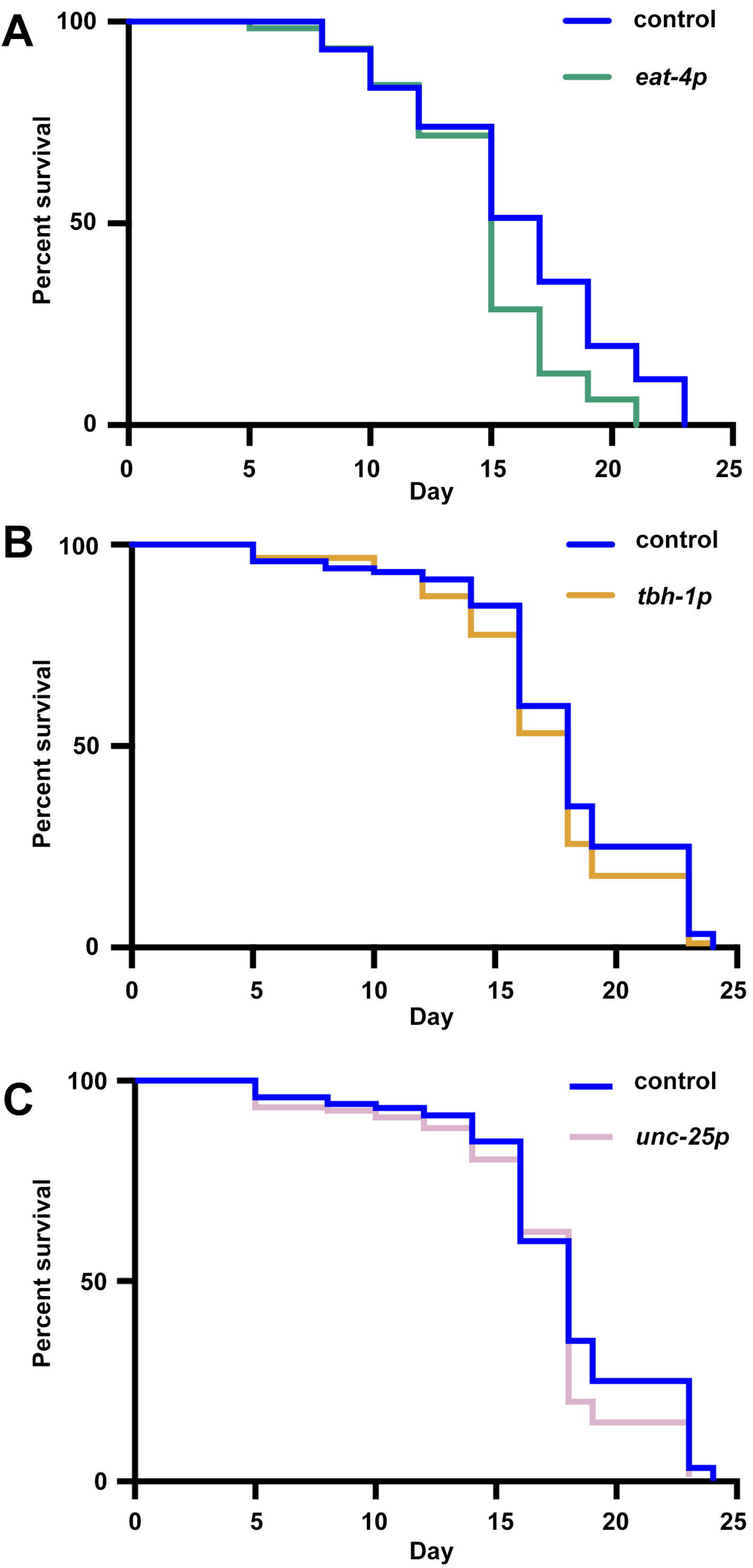

A
control
eat-4p
Percent survival
100
50
0
0
5
10
15
20
25
Day
B
control
tbh-1p
Percent survival
100
50
0
0
5
10
15
20
25
Day
C
control
unc-25p
Percent survival
100
50
0
0
5
10
15
20
25
Day

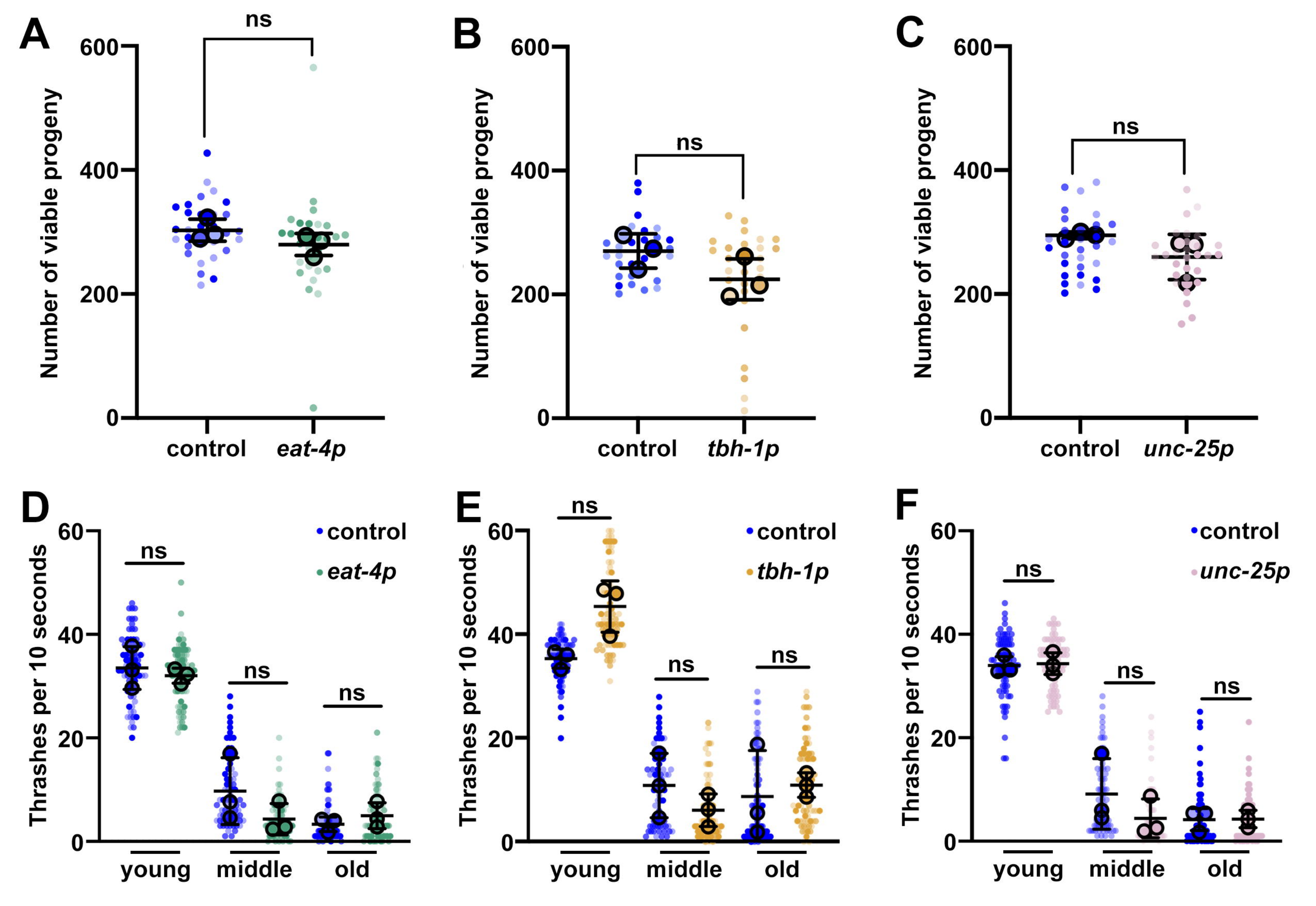

A
B
C
D
E
F
Number of viable progeny
600
400
200
0
ns
control
eat-4p
tbh-1p
unc-25p
Thrashes per 10 seconds
60
40
20
0
control
eat-4p
tbh-1p
unc-25p
young
middle
old

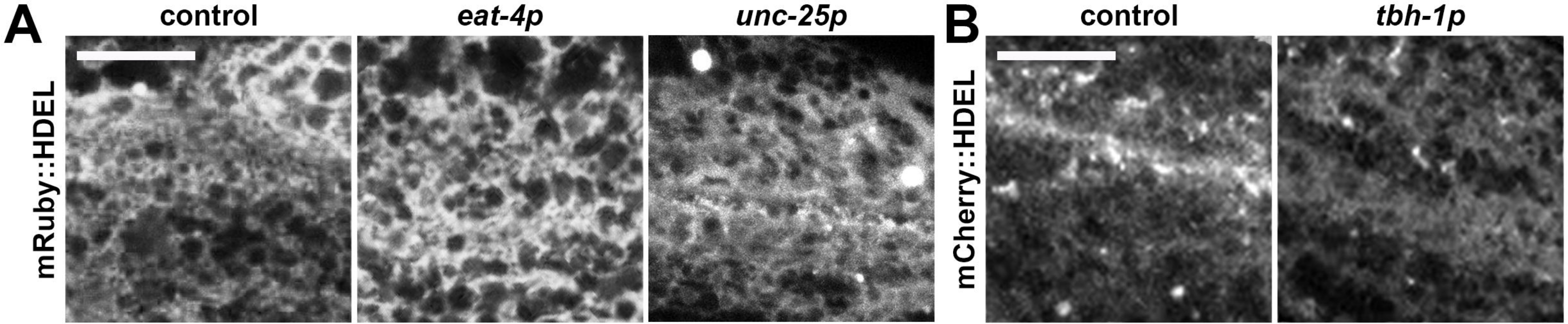
A
control
eat-4p
unc-25p
mRuby::HDEL
B
control
tbh-1p
mCherry::HDEL

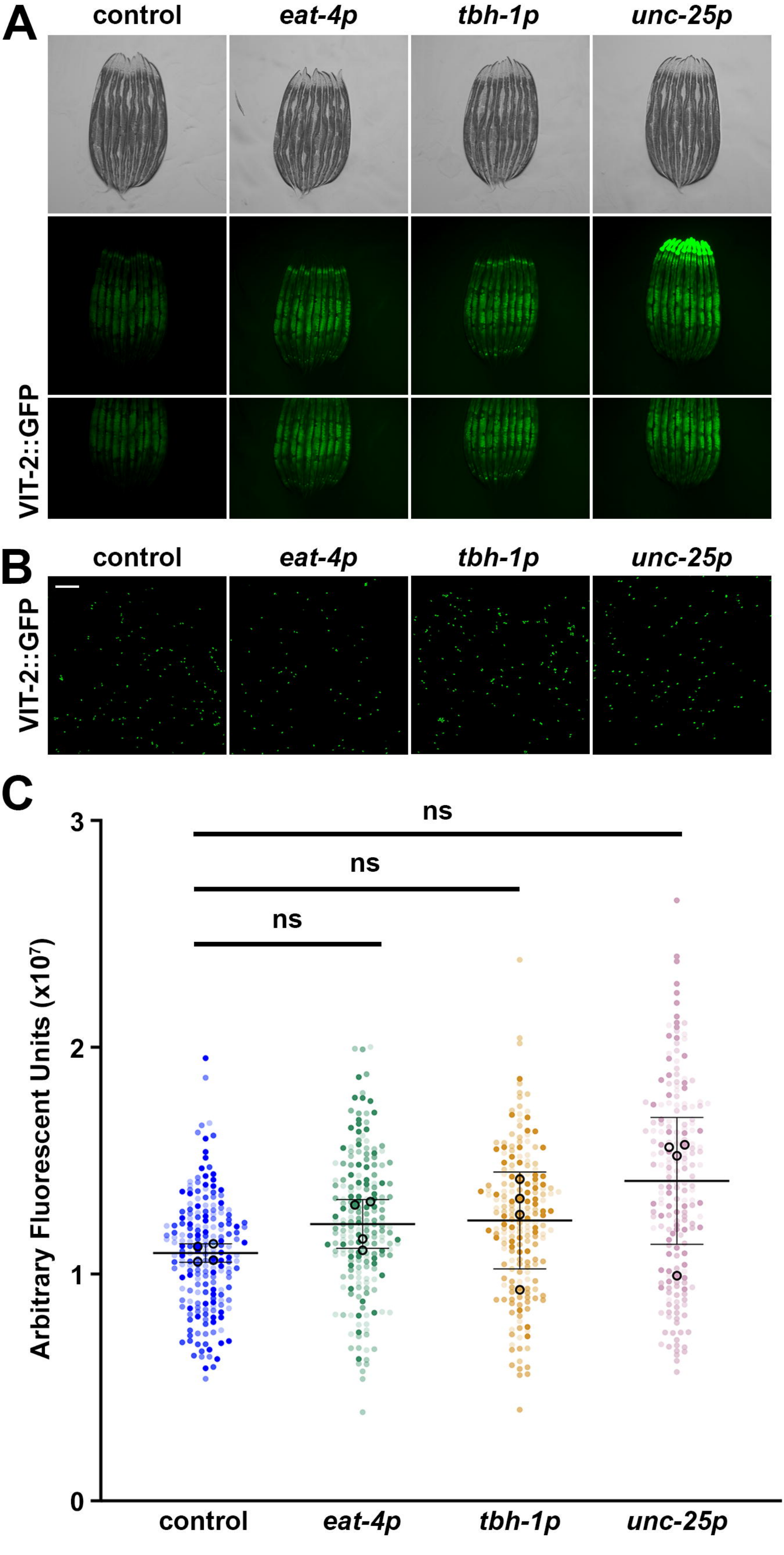

A
control
eat-4p
tbh-1p
unc-25p
VIT-2::GFP
B
control
eat-4p
tbh-1p
unc-25p
VIT-2::GFP
C
ns
ns
ns
Arbitrary Fluorescent Units (x10^7)
3
2
1
0
control
eat-4p
tbh-1p
unc-25p
